# Supplementary material for: Vibrio cholerae’s ToxRS bile sensing system
Source: eLife. 2023 Sep 28;12:e88721. doi: 10.7554/eLife.88721 (PMC10624426; doi:10.7554/eLife.88721)
Supplement: Figure 4—source data 1. [file elife-88721-fig4-data1.zip › MMS_source_data/AnalysisReport_20230505_071110Z.pdf]

RedShift AQS³ delta Analytics Results

Report Date:	Friday, May 5, 2023 9:11:10 AM
Report File:	"AnalysisReport_20230505_071110Z.pdf"
Username:	Default UserEx
Version(s):	GUI: 2.5.98.4131
DataSet Name:	"20221122_Uni_Graz"
Proc. Data Folder:	"pdata_Evaluation report_20221124_114232Z"
Processed Date:	Thursday, November 24, 2022 12:42:32 PM

Table of Analyzed Sample Data:

Clr	Included?	Collection Type	Replicate	Sample Name
	Yes	Sample/Buffer (SB)	(avg)	RS_1.Buffer 1
	Yes	Sample/Buffer (SB)	(avg)	RS_2.Buffer 2
	Yes	Sample/Buffer (SB)	(avg)	RS_3.Buffer 3
	Yes	Model Protein		NIST mAb

Table of Sample Replicates in Averaging:

Clr	Included?	Collection Type	Replicate	Sample Name
	Yes	Sample/Buffer (SB)	01	RS_1.Buffer 1
	Yes	Sample/Buffer (SB)	02	RS_1.Buffer 1
	Yes	Sample/Buffer (SB)	03	RS_1.Buffer 1
	Yes	Sample/Buffer (SB)	01	RS_2.Buffer 2
	Yes	Sample/Buffer (SB)	02	RS_2.Buffer 2
	Yes	Sample/Buffer (SB)	03	RS_2.Buffer 2
	Yes	Sample/Buffer (SB)	01	RS_3.Buffer 3
	Yes	Sample/Buffer (SB)	02	RS_3.Buffer 3
	Yes	Sample/Buffer (SB)	03	RS_3.Buffer 3

Table of Water and Buffer Replicates:

Included?	Collection Type	Replicate	Sample Name
Yes	Water/Water (WW)	01	Water
Yes	Water/Water (WW)	02	Water
Yes	Water/Water (WW)	03	Water
Yes	Buffer/Water (BW)	01	Buffer 1.Water
Yes	Buffer/Water (BW)	02	Buffer 1.Water
Yes	Buffer/Water (BW)	03	Buffer 1.Water
Yes	Buffer/Buffer (BB)	01	Buffer 1
Yes	Buffer/Buffer (BB)	02	Buffer 1
Yes	Buffer/Buffer (BB)	03	Buffer 1
Yes	Buffer/Water (BW)	01	Buffer 2.Water
Yes	Buffer/Water (BW)	02	Buffer 2.Water
Yes	Buffer/Water (BW)	03	Buffer 2.Water
Yes	Buffer/Buffer (BB)	01	Buffer 2
Yes	Buffer/Buffer (BB)	02	Buffer 2
Yes	Buffer/Buffer (BB)	03	Buffer 2
Yes	Buffer/Water (BW)	01	Buffer 3.Water
Yes	Buffer/Water (BW)	02	Buffer 3.Water
Yes	Buffer/Water (BW)	03	Buffer 3.Water
Yes	Buffer/Buffer (BB)	01	Buffer 3
Yes	Buffer/Buffer (BB)	02	Buffer 3
Yes	Buffer/Buffer (BB)	03	Buffer 3

Graph Results: Raw Diff AU

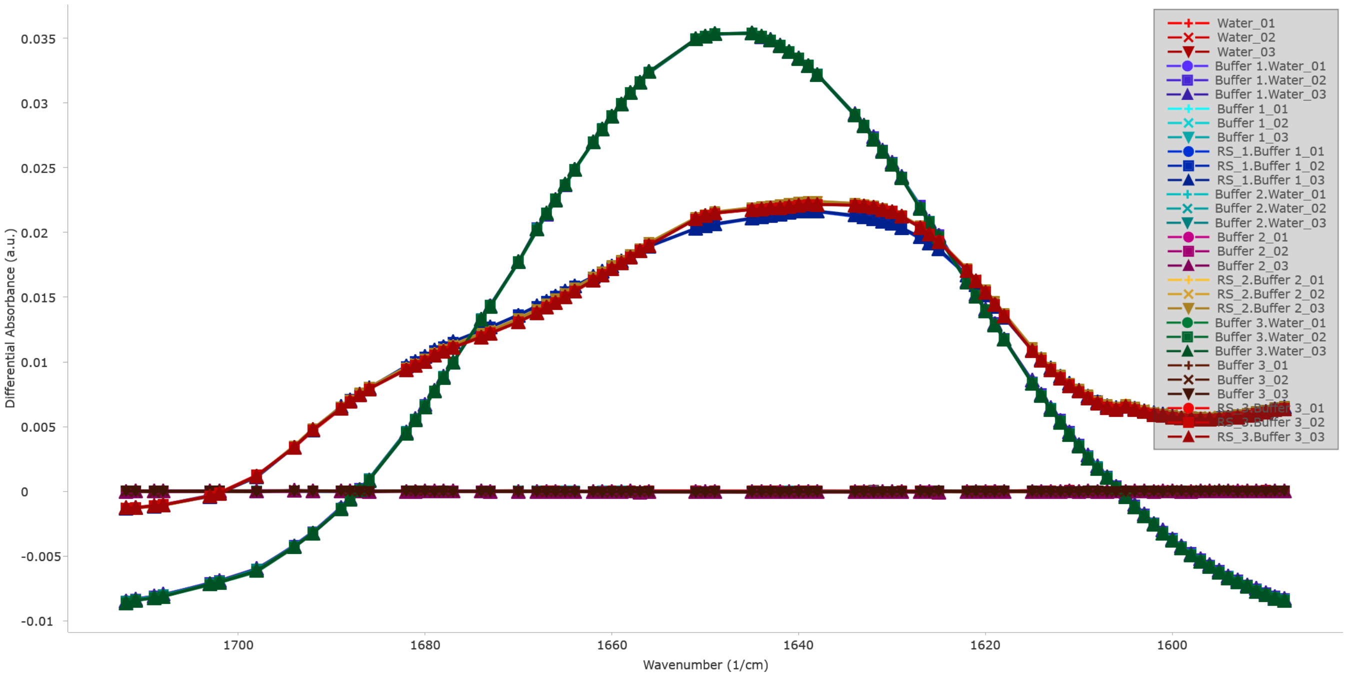

Graph Results: Absolute AU

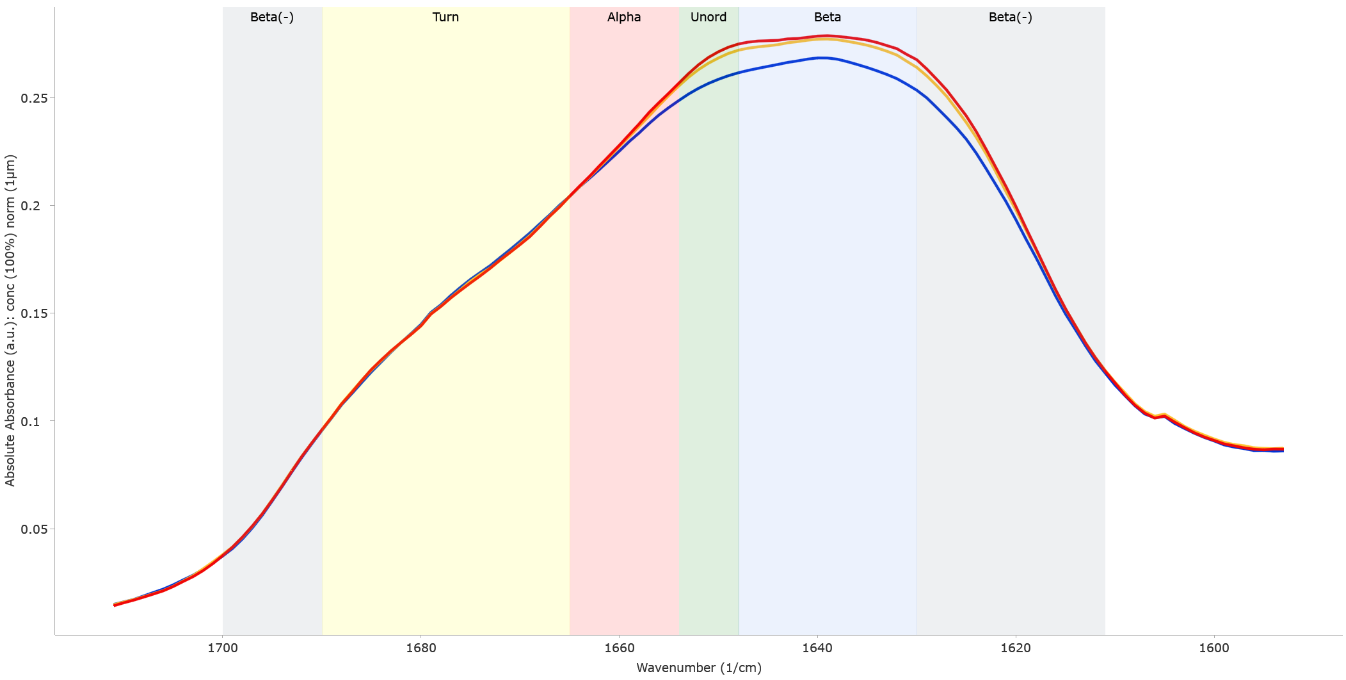

Analysis Settings for Absolute Spectrum:

Cell Pathlength(µm):	23.50
Water Data:	"water spectra"
Normalization	
100% Conc:	True
1 µm Path:	True
Spacing(1/cm):	1.0
Interpolation:	Spline

Graph Results: Second Derivative

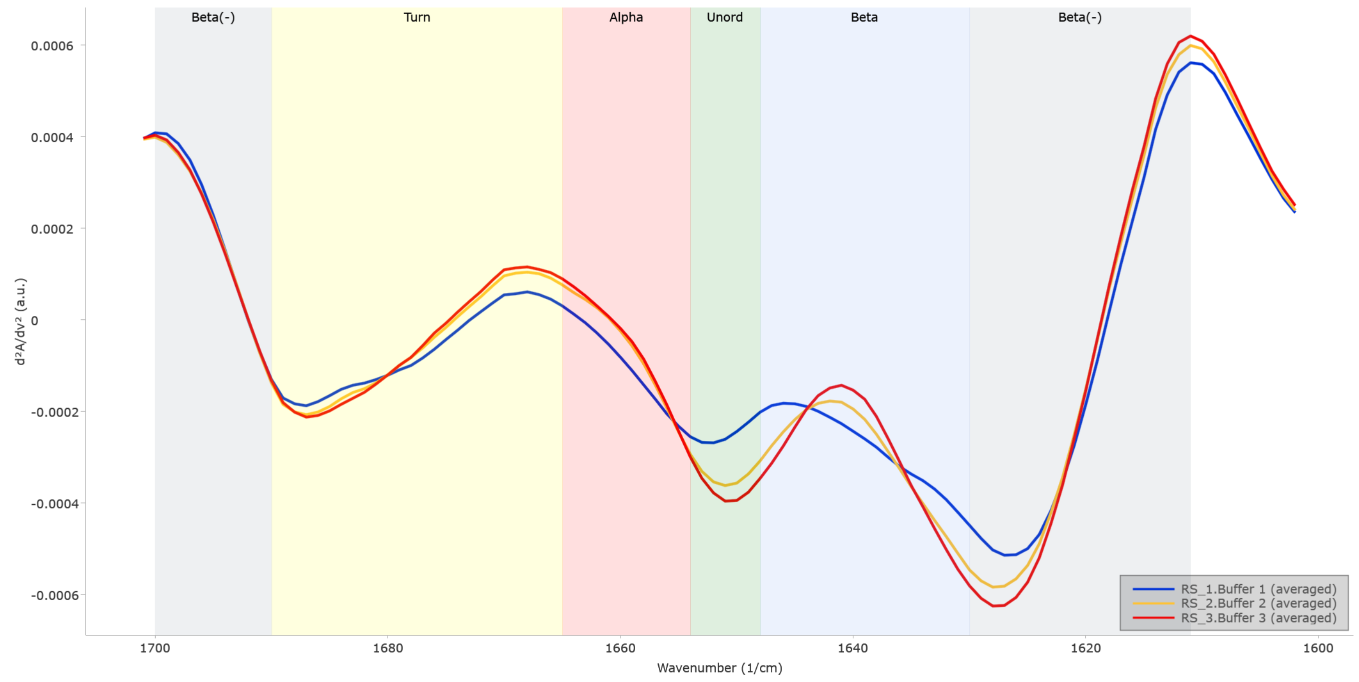

Analysis Settings for Second Derivative:

Normalization: False
Smoothing Window Size
Sample/Model: 19

Graph Results: Similarity (Area of Overlap)

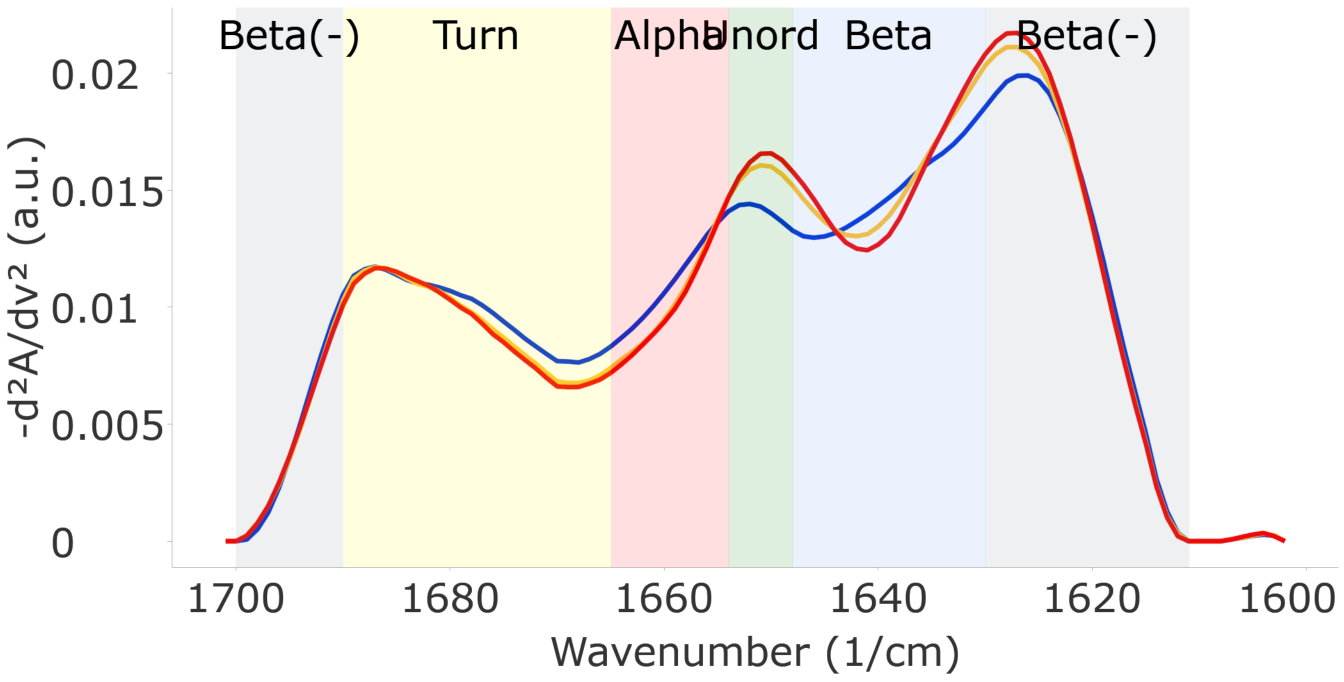

Analysis Settings for Similarity (Area of Overlap):

Spectral Range:	Full Data Range
Baseline:	Rubberband
Normalization:	Normalize to Area
Comparison:	Mean/Selected
Include Model Protein:	False

Graph of Area of Overlap and Comparison Set:

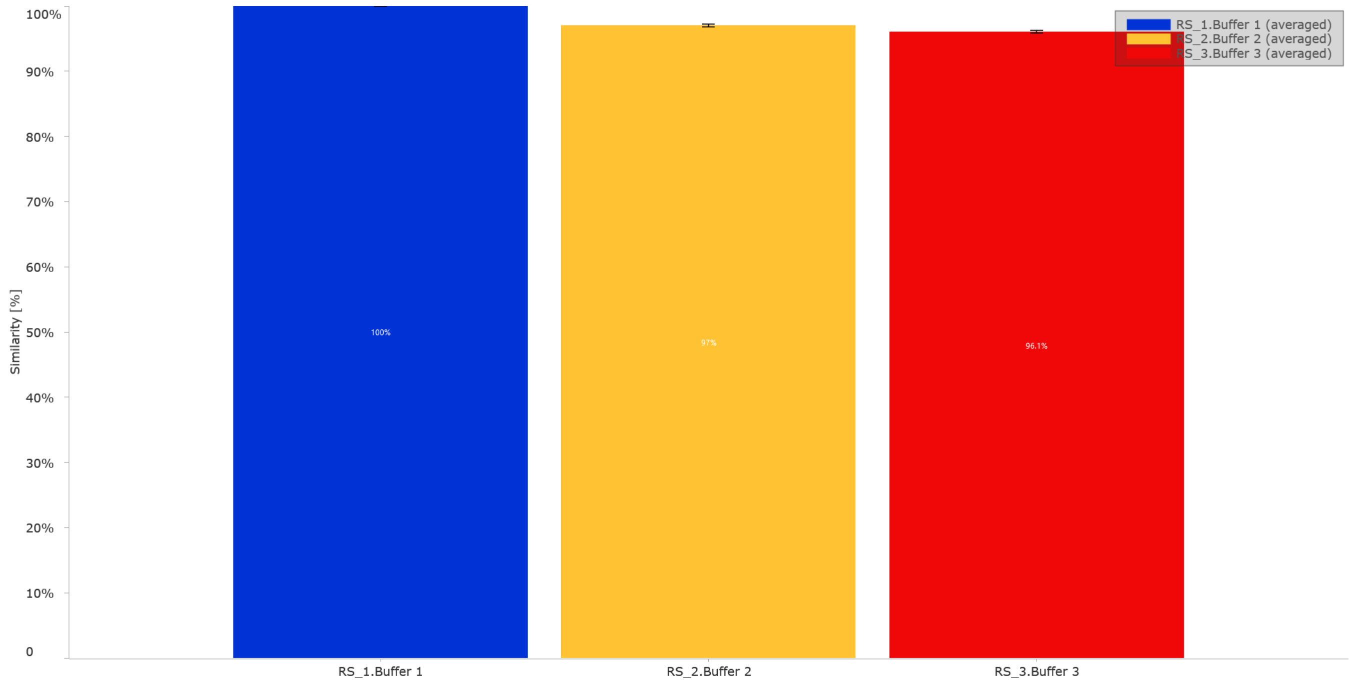

Table of Area of Overlap and Comparison Set:

AO(%)	CompareSet	Replicate	Sample Name
100.0	Yes	(avg)	RS_1.Buffer 1
97.0	No	(avg)	RS_2.Buffer 2
96.1	No	(avg)	RS_3.Buffer 3
72.5	No		NIST mAb

Similarity (Area of Overlap) Replicate Summary Table:

Conc (mg/mL)	Mean±SD	Repeat.	AO (%) of Replicates	Sample Name
5.00	99.72±0.04	99.72	99.8, 99.7, 99.7	RS_1.Buffer 1
5.00	96.92±0.21	99.54	96.7, 97.0, 97.1	RS_2.Buffer 2
5.00	96.02±0.20	99.56	95.9, 95.9, 96.2	RS_3.Buffer 3

Graph Results: Similarity (Weighted Spectral Difference)

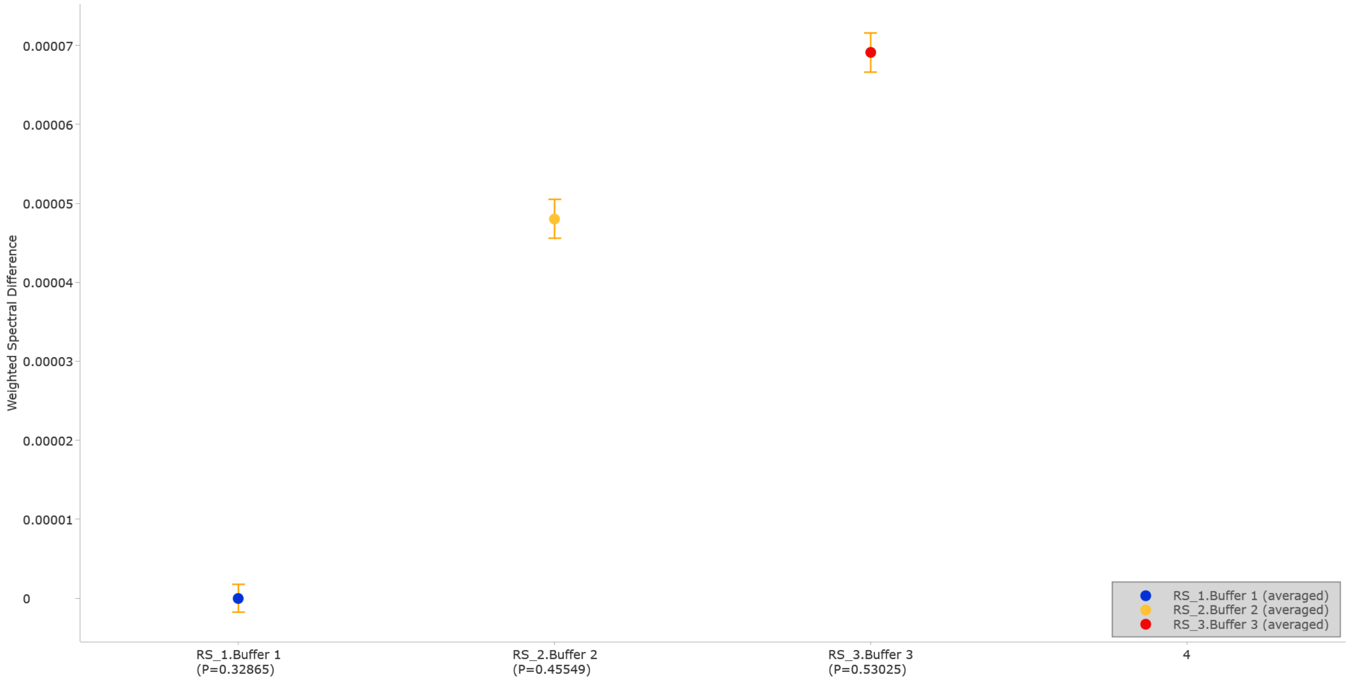

Analysis Settings for Similarity (Weighted Spectral Difference):

Data Used:	2nd Derivative
Comparison:	Mean/Selected

Table of Weighted Spectral Difference and Comparison Set:

WSD	CompareSet	Replicate	Sample Name
0	Yes	(avg)	RS_1.Buffer 1
4.805e-05	No	(avg)	RS_2.Buffer 2
6.9098e-05	No	(avg)	RS_3.Buffer 3
0.00052173	No		NIST mAb

Area of Overlap and Weighted Spectral Difference Summary Table

Sample	AO Compare Set?	AO(%)	WSD Compare Set?	WSD	P Value
RS_1.Buffer 1_(avg)	Yes	100.0	Yes	0	0.32865
RS_3.Buffer 3_(avg)	No	96.1	No	6.9098e-05	0.53025
RS_2.Buffer 2_(avg)	No	97.0	No	4.805e-05	0.45549
NIST mAb	No	72.5	No	0.00052173	N/A

Graph Results: Delta

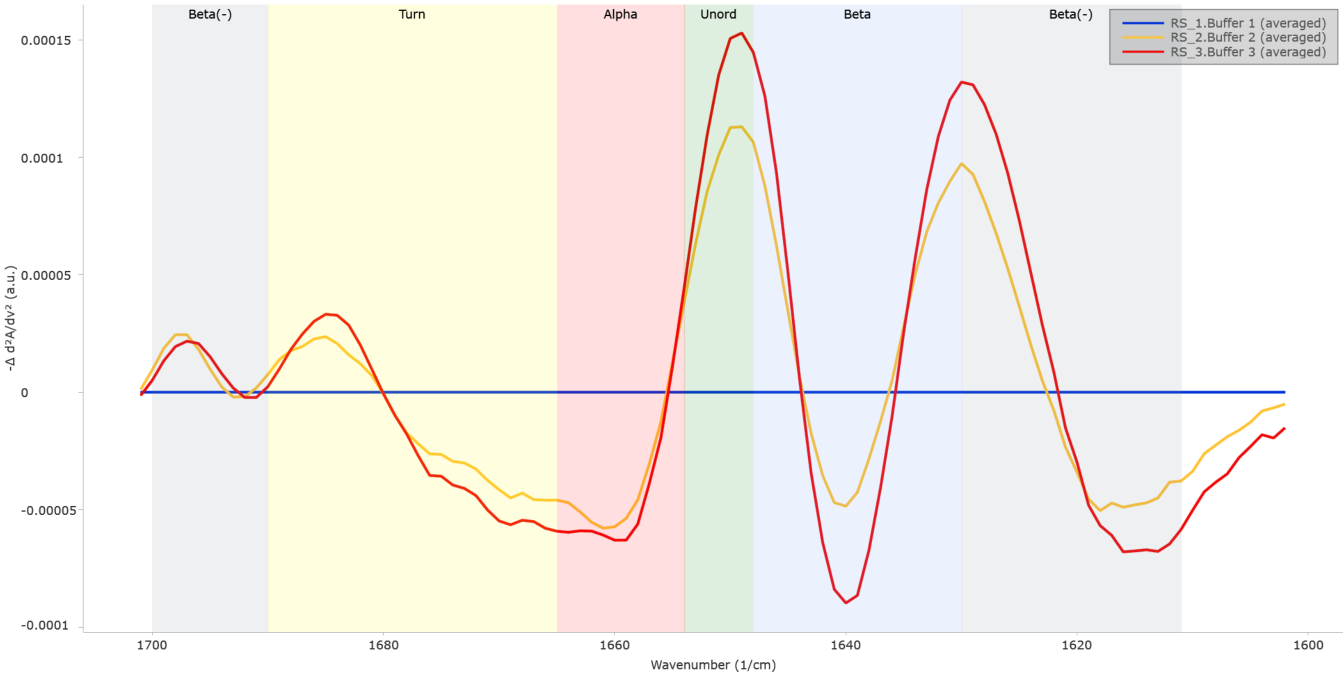

Analysis Settings for Delta:

Use 2ndDeriv:	True
Comparison:	Mean/Selected
Include Model Protein:	False
Min/Max Delta Range:	0.0000000E+000 to 0.0000000E+000

Table of Delta Bounds and Comparison Set:

Within Min/Max Bounds	CompareSet	Replicate	Sample Name
N/A	Yes	(avg)	RS_1.Buffer 1
N/A	No	(avg)	RS_2.Buffer 2
N/A	No	(avg)	RS_3.Buffer 3
N/A	No		NIST mAb

Graph Results: Stability

Data used for Stability: Delta

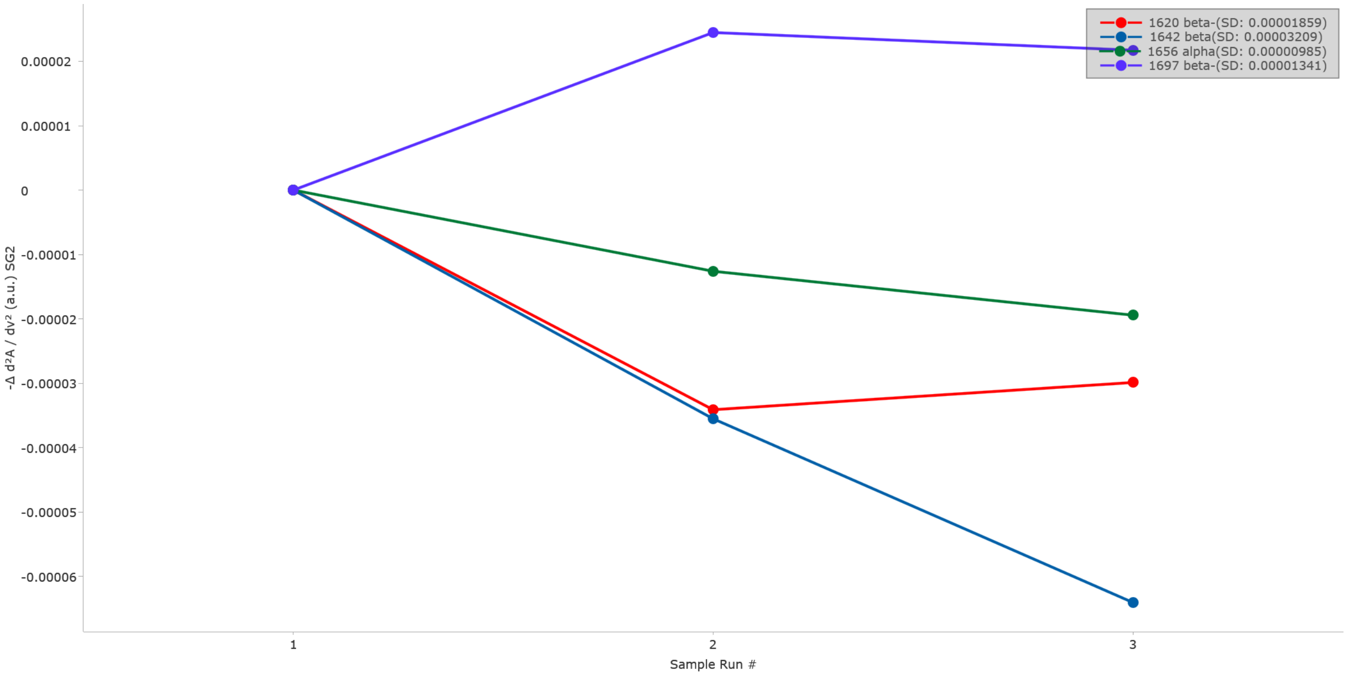

Graph Results: Quantitation

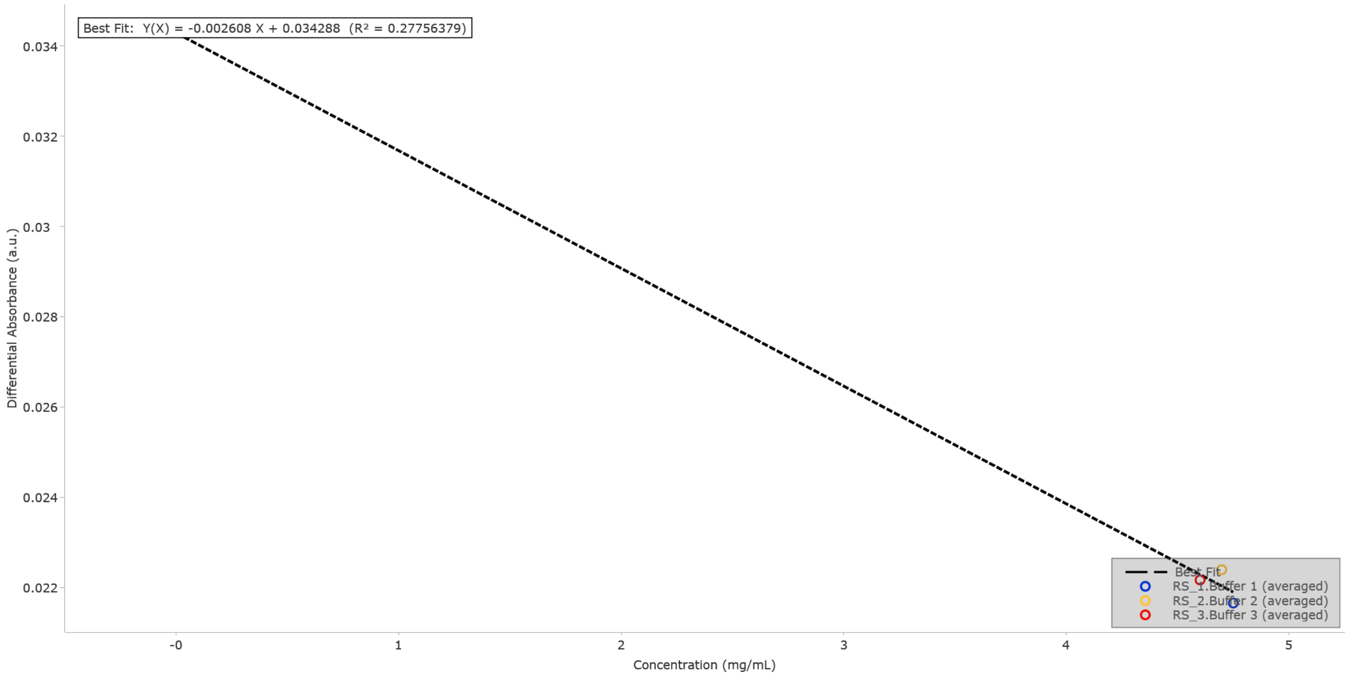

Analysis Settings for Quantitation:

Model Protein: NIST mAb
Concentration Plotted: Fit

Table of Concentrations and Displacement Factors:

Conc (mg/mL)	Fit Conc(mg/mL)	Fit DF	Replicate	Sample Name
5.00	4.75	0.6000	(avg)	RS_1.Buffer 1
5.00	4.70	0.6000	(avg)	RS_2.Buffer 2
5.00	4.60	0.5940	(avg)	RS_3.Buffer 3

Graph Results: Gaussian Fit

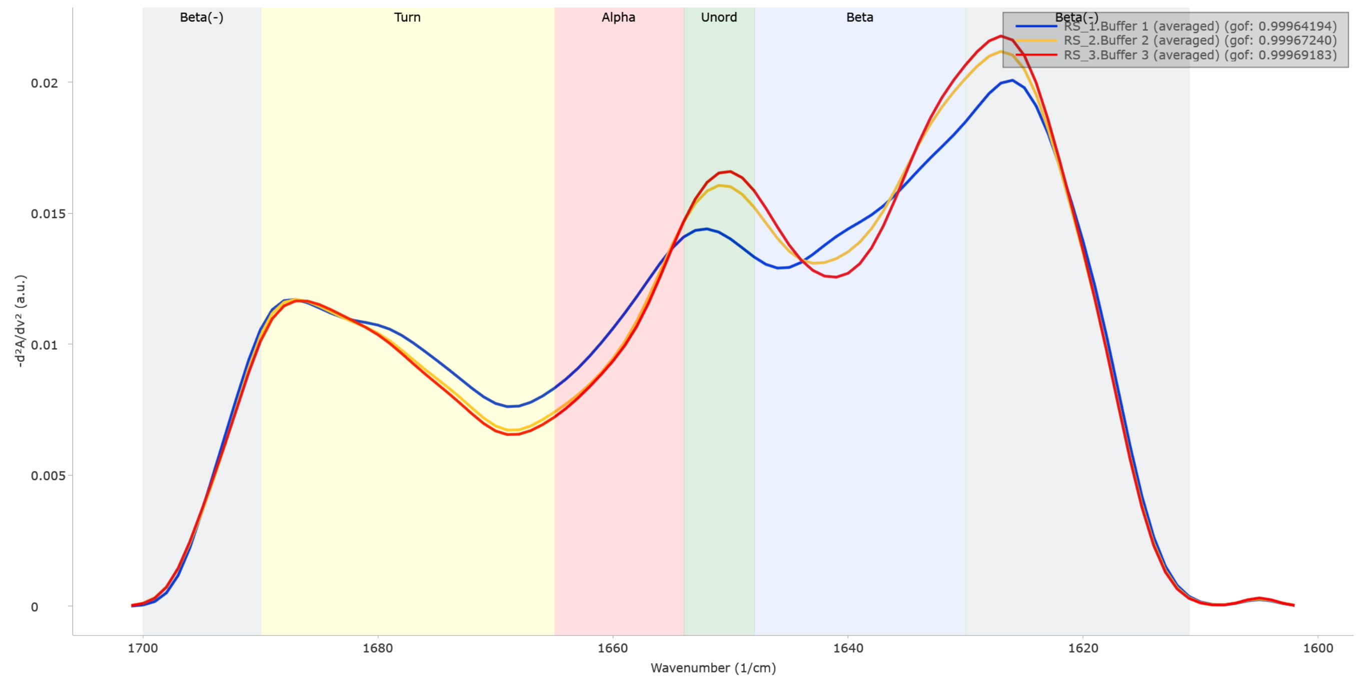

Table of Gaussian Fit R^2:

Gaussian Fit R^2 Value	Replicate	Sample Name
0.99964	(avg)	RS_1.Buffer 1
0.99967	(avg)	RS_2.Buffer 2
0.99969	(avg)	RS_3.Buffer 3

Table of Gaussian Term Settings (*default):

Wavelength(1/cm)	Structure	PeakWindow(+/-)	FWHM(1/cm)
1597.0	side	1.0 *	10 *
1610.0	beta-	-5.0 to 0.0 *	6 *
1618.0	beta-	3.0 *	6 *
1626.0	beta-	1.0 *	6 *
1632.0	beta	1.0 *	6 *
1642.0	beta	1.0 *	6 *
1650.0	unord	1.0 *	6 *
1656.0	alpha	1.0 *	6 *
1660.0	turn	1.0 *	6 *
1666.0	turn	1.0 *	6 *
1672.0	turn	1.0 *	6 *
1680.0	turn	1.0 *	6 *
1688.0	turn	1.0 *	6 *
1690.0	beta	1.0 *	6 *
1695.0	beta-	1.0 *	6 *
1704.0	side	1.0 *	20 *

Graph Results: HOS (Structure)

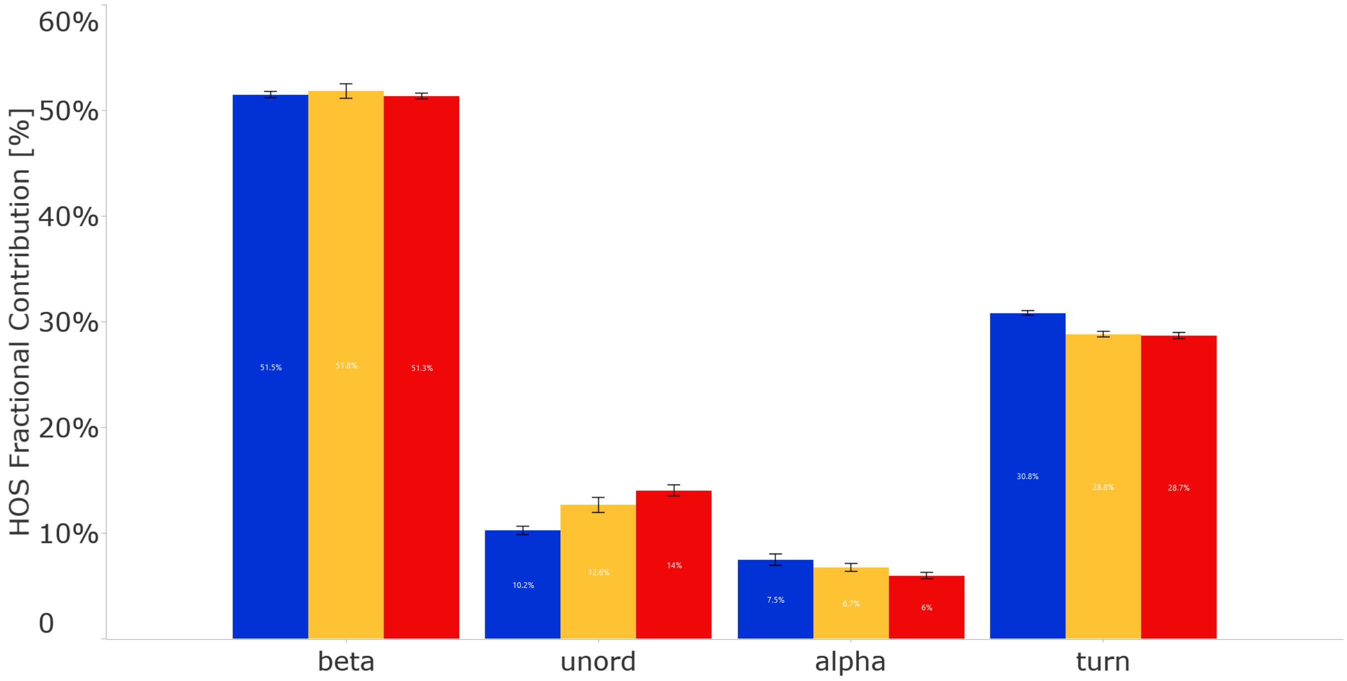

Table of HOS Statistics:

HOS Bin Name	Fractional(%) Mean	Fractional(%) StdDev
beta	51.5	0.24
unord	12.3	1.92
alpha	6.7	0.75
turn	29.4	1.20

High Order Structure (HOS) Replicate Summary Table:

Conc (mg/mL)	beta Mean±SD	unord Mean±SD	alpha Mean±SD	turn Mean±SD	Sample Name
5.00	51.7±0.30	10.2±0.40	7.4±0.54	30.7±0.22	RS_1.Buffer 1
5.00	52.0±0.69	12.4±0.71	6.7±0.38	28.9±0.26	RS_2.Buffer 2
5.00	51.6±0.27	13.9±0.53	5.9±0.30	28.6±0.29	RS_3.Buffer 3
