# Supplementary material for: Vibrio cholerae’s ToxRS bile sensing system
Source: eLife. 2023 Sep 28;12:e88721. doi: 10.7554/eLife.88721 (PMC10624426; doi:10.7554/eLife.88721)
Supplement: Supplementary file 1. — (a) Description of expression constructs. List of plasmids and amino acid sequences used for the expression of ToxRp and ToxSp. (b) Crystal data and structure refinement table of ToxRSp. (c) Analysis of size-exclusion chromatography coupled with solution small-angle X-ray scattering SEC-SAXS. According to SAXS curves, ToxRp is monomeric in solution, whereas ToxSp seems to form dimers. Upon bile addition, the radius of gyration RG, the maximum particle size Dmax and the molecular weight of the ToxRSp complex increases. (d) NMR derived dissociation constants for ToxRp and sodium cholate hydrate. NMR titration experiments reveal a weak binding of bile to ToxRp with a dissociation constant of 2.6 mM. Table S5 includes ToxRp residue number, chemical shift distance, dissociation constant Kd [M], error of Kd. The calculated average Kd is 2.7±1.4 mM. (e) List of amino acid sequences of sensory domains of ToxRS proteins from different Vibrio species. Table S5 includes UniProt ID of the used sequences as well as sequence identity values to ToxRSp from V. cholerae. (f) Structural homology search of ToxSp bound to ToxRp using Dali server (Holm, 2022). [file elife-88721-supp1.docx]

**Supplementary File 1**

*Vibrio cholerae's* ToxRS Bile Sensing System

N. Gubensäk^1*^, T. Sagmeister^1^, C. Buhlheller^1^, B. D. Geronimo^2^, G. E. Wagner^3,4^, L. Petrowitsch^1^, M. Gräwert^5^, M. Rotzinger^3^, T. M. I. Berger^1^, J. Schäfer^6^, I. Usón^7,8^, J. Reidl^1,9,10^, P. A. Sánchez-Murcia^2^, K. Zangger^3,9,10^ and T. Pavkov-Keller^1,9,10^*

1 Institute of Molecular Biosciences; University of Graz, Graz, Austria.

2 Laboratory of Computer-Aided Molecular Design, Division of Medicinal Chemistry, Otto-Loewi Research Center; Medical University of Graz, Graz, Austria.

3 Institute of Chemistry / Organic and Bioorganic Chemistry; University of Graz, Graz, Austria.

4 Diagnostic and Research Institute of Hygiene, Microbiology and Environmental Medicine, Medical University of Graz, Graz, Austria.

5 EMBL Hamburg; Hamburg, Germany.

6 RedShiftBio; Boxborough, Massachusetts, United States.

7 Institute of Molecular Biology of Barcelona (IBMB–CSIC); Barcelona, Spain.

8 ICREA, Institució Catalana de Recerca i Estudis Avançats; Barcelona, Spain.

9 BioHealth Field of Excellence, University of Graz; Graz, Austria.

10 BioTechMed-Graz; Graz, Austria..

*Nina Gubensäk, Tea Pavkov-Keller

Email: nina.gubensaek@uni-graz.at, tea-pavkov@uni-graz.at

| Protein | Vector | N-terminal 6xHis Tag and amino acid sequence |
| --- | --- | --- |
| ToxRp | pQE30 | MRGSHHHHHHGSPSQTSFKPLTVVDGVAVNMPNNHPDLSNWLPSIELCVKKYNEKHTGGLKPIEVIATGGQNNQLTLNYIHSPEVSGENITLRIVANPNDAIKVCE |
| ToxSp | pCDFDuet | MGSSHHHHHHSQDPNSDFKLEQVLTSREWQSKMVSLIKTNSNRPAMGPLSRVDVTSNVKYLPNGTYLRVSIVKLFSDDNSAESVINISEFGEWDISDNYLLVTPVEFKDISSNQSKDFTDEQLQLITQLFKMDAQQSRRVDIVNERTILFTSLSHGSTVLFSNS |

**Supplementary File 1a.**

**Description of expression constructs.** List of plasmids and amino acid sequences used for the expression of ToxRp and ToxSp.

**ToxRSp crystal structure**

| Resolution range | 49.27 - 3.002 (3.109 - 3.002) |
| --- | --- |
| Space group | P 6_5_ |
| Unit cell (Å, °) | 72.507, 72.507, 79.463  90, 90, 120 |
| Total reflections | 9392 (909) |
| Unique reflections | 4786 (467) |
| Multiplicity | 2.0 (1.9) |
| Completeness (%) | 99.85 (100.00) |
| Mean I/sigma(I) | 13.10 (2.18) |
| Wilson B-factor | 75.07 |
| R-merge | 0.03888 (0.3675) |
| R-meas | 0.05498 (0.5197) |
| R-pim | 0.03888 (0.3675) |
| CC1/2 | 0.999 (0.591) |
| CC* | 1 (0.862) |
| Reflections used in refinement | 4785 (467) |
| Reflections used for R-free | 260 (38) |
| R-work | 0.2163 (0.2886) |
| R-free | 0.2491 (0.3463) |
| CC(work) | 0.940 (0.656) |
| CC(free) | 0.884 (0.774) |
| Number of non-hydrogen atoms | 1865 |
| Macromolecules | 1854 |
| Ligands | 0 |
| Solvent | 11 |
| Protein residues | 236 |
| RMS(bonds) | 0.012 |
| RMS(angles) | 1.86 |
| Ramachandran favoured (%) | 93.53 |
| Ramachandran allowed (%) | 6.47 |
| Ramachandran outliers (%) | 0.00 |
| Rotamer outliers (%) | 0.92 |
| Clash score | 4.32 |
| Average B-factor | 87.41 |
| Macromolecules | 87.60 |
| Solvent | 55.04 |

**Supplementary File 1b.**

**Crystal data and structure refinement table of ToxRSp.**

|  | **Radius of Gyration,**  **R_G_ [nm]** | **Dmax**  **[nm]** | **Chi²** | **MW estimation [kDa]** | | |
| --- | --- | --- | --- | --- | --- | --- |
|  |  |  |  | MoW | VC | Bayesian |
| ToxRp | 1.6 +/- 0.1 | 6.0 +/- 0.2 | 1.6 | 9 +/- 1 | 11 +/- 1 | 10 +/- 1 |
| ToxSp (dimeric) | 2.3 +/- 0.1 | 7.4 +/- 0.2 | 1.4 | 33 +/- 3 | 35 +/- 3 | 35 +/- 3 |
| ToxRSp | 2.1 +/- 0.1 | 6.6 +/- 0.2 | 1.1 | 30 +/- 3 | 28 +/- 3 | 28 +/- 3 |
| ToxRSp + bile (1:1) | 2.4+/- 0.1 | 7.6 +/- 0.5 | 1.9 | 32 +/- 3 | 29 +/- 3 | 29 +/- 3 |

**Supplementary File 1c.**

**Analysis of size-exclusion chromatography coupled with solution small-angle X-ray scattering SEC-SAXS.** According to SAXS curves, ToxRp is monomeric in solution, whereas ToxSp seems to form dimers. Upon bile addition, the radius of gyration R_G_, the maximum particle size D_max_ and the molecular weight of the ToxRSp complex increases.

| **Residue Nr** | **Shift Dist** | **Kd [M]** | **Kd error** | **average Kd [M]** | **averaged Kd error** |
| --- | --- | --- | --- | --- | --- |
| 206 | 0.02682 | 0.00125 | 0.00138 | **0.0026412** | **0.0014211** |
| 209 | 0.0113 | 0.00202 | 0.00333 |  |  |
| 210 | 0.01169 | 0.00247 | 0.00234 |  |  |
| 211 | 0.01454 | 0.00182 | 0.00144 |  |  |
| 212 | 0.0016 | 0.00286 | 0.00282 |  |  |
| 214 | 0.00901 | 0.00219 | 0.00154 |  |  |
| 215 | 0.00701 | 0.00076 | 0.00085 |  |  |
| 216 | 0.03678 | 0.00172 | 0.00254 |  |  |
| 217 | 0.00916 | 0.00165 | 0.00267 |  |  |
| 218 | 0.01834 | 0.0026 | 0.00492 |  |  |
| 219 | 0.01981 | 0.0062 | 0.00921 |  |  |
| 221 | 0.03636 | 0.00327 | 0.00578 |  |  |
| 223 | 0.01701 | 0.00223 | 0.00284 |  |  |
| 226 | 0.01856 | 0.0009 | 0.00148 |  |  |
| 227 | 0.00908 | 0.00184 | 0.00255 |  |  |
| 229 | 0.00989 | 0.00233 | 0.00288 |  |  |
| 233 | 0.00806 | 0.0028 | 0.00415 |  |  |
| 234 | 0.00461 | 0.00358 | 0.00587 |  |  |
| 235 | 0.00753 | 0.00195 | 0.00191 |  |  |
| 237 | 0.04098 | 0.0015 | 0.00234 |  |  |
| 243 | 0.01332 | 0.00492 | 0.00589 |  |  |
| 251 | 0.04908 | 0.00164 | 0.00283 |  |  |
| 252 | 0.04201 | 0.00273 | 0.00154 |  |  |
| 253 | 0.01824 | 0.00184 | 0.00217 |  |  |
| 254 | 0.00861 | 0.00166 | 0.00194 |  |  |
| 255 | 0.00993 | 0.00213 | 0.00311 |  |  |
| 256 | 0.03031 | 0.00217 | 0.00325 |  |  |
| 262 | 0.00986 | 0.00597 | 0.01658 |  |  |
| 263 | 0.02958 | 0.0013 | 0.00223 |  |  |
| 264 | 0.0437 | 0.00226 | 0.00439 |  |  |
| 265 | 0.02948 | 0.00606 | 0.0069 |  |  |
| 269 | 0.04066 | 0.00431 | 0.00027 |  |  |
| 270 | 0.02556 | 0.00423 | 0.00534 |  |  |

**Supplementary File 1d.**

**NMR derived dissociation constants for ToxRp and sodium cholate hydrate.** NMR titration experiments reveal a weak binding of bile to ToxRp with a dissociation constant of 2.6 mM. Table S5 includes ToxRp residue number, chemical shift distance, dissociation constant Kd [M], error of Kd. The calculated average Kd is 2.7 ± 1.4 mM.

| **Organism** | **Protein** | **UniProt** | **AA sequence** | **Identity to V.c. %** |
| --- | --- | --- | --- | --- |
| *V. cholerae* | ToxRp | P24003 | TNPSQTSFKPLTVVDGVAVNMPNNHPDLSNWLPSIELCVKKYNEKHTGGLKPIEVIATGGQNNQLTLNYIHSPEVSGENITLRIVANPNDAIKVCE | 100 |
|  | ToxSp | P15795 | DFKLEQVLTSREWQSKMVSLIKTNSNRPAMGPLSRVDVTSNVKYLPNGTYLRVSIVKLFSDDNSAESVINISEFGEWDISDNYLLVTPVEFKDISSNQSKDFTDEQLQLITQLFKMDAQQSRRVDIVNERTILFTSLSHGSTVLFSNS | 100 |
| *V. vulnificus* | ToxRp | Q7MMR8 | PSESKFRLLENVNGVEVLTPLNHPPLQAWMPSIRQCVNKYAETHTGDSAPVKVIATGGQGNQLILNYIHTLPHSNENVTLRIFSEQNDLGSICK | 54.26 |
|  | ToxSp | Q9RP85 | DLKVEQVLTSKEWQSYMVTVITDDLQNEGSVGPLRKVTLTSNVKYLPNGNYVRVSVLKLYSNESSQEVVINISESGTWDVSDNYLLVTAKEFKDISSSQSKDFSDAQLKLITKVFKMDAQQSRRIDIVNDQTLLLTSLNHGSSVLFSN | 68.71 |
| *V. parahaemolyticus* | ToxRp | Q05938 | NPAESQFRQIGEYQNVPVMTPVNHPQINNWLPSIEQCIERYVKHHAEDSLPVEVIATGGQNNQLILNYIHDSNHSYENVTLRIFAGQNDPTDICK | 50.53 |
|  | ToxSp | Q05939 | DLKVEQVLTSNEWQSTMVTVITDNLPDDTVGPLRRVNVESNVKYLPNGDYIRVANIKLFAQGSTAESTINISEKGRWEVSDNYLLVSPSEFKDISSSQSKDFSEAQLRLITQIFKLDAEQSRRIDVVNEKTLLLTSLNHGSTVLFRN | 67.12 |
| *V. mimicus* | ToxRp | A7LAQ0 | KFKPLTVVDDVAVNMPINHPDLSNWLPSIELCVKKYNEKHTGGLKPVEVIATGGQNNQLTLNYIHSPEVSGENITLRIVANPTDAVKVCE | 93.33 |
|  | ToxSp | A7LAQ1 | KLEQVLTSREWQSKMVSLIKTKSNSPAMGPLSRVDVSSNVKYLPNGTYLRVSIVKLYSDGNSAESTINISESGEWDISDNYLLVTPIQFKDISSNQSKDFTDEQLQLITQLFKMDAQQSRRVDIVNERTILFTSLNHGSTVLFSNS | 93.15 |
| *V. diabolicus* | ToxRp | A0A6V7GR35 | AESQFRQVGEYQNVPVMTPVNHPQINSWLPSIEQCIERYVKYHAEDSMPVEVIATGGQNNQLILNYIHDSQHSYENVTLRIFAGQNDPTDICK | 48.39 |
|  | ToxSp | A0A6G9WH38 | DLKVEQLLTANEWQSTMVTVITDSLPDDTVGPLRRVNVESNVKYLPNGDYIRVANIKLFAQGSTAESTINISEKGRWEVSDNYLLVSPSEFKDISSSQSKDFSEAQLRLITQIFKLDAEQSRRIDVVNEKTLLLTSLNHGSSVLFKN | 64.38 |
| *V. alginolyticus* | ToxRp | A0A060A4C9 | ASNTINWLPRVIIFLSLLLPVCVLLFTNPAESQFRQIGEYQSVPVMTPVNHPQINNWLPSIEQCIERYVKHHAEDSLPVEVIATGGQDNQLILNYIHDSNHSYENVTLRIFAGQNDPTDIC | 50.53 |
|  | ToxSp | A0A6F8W9U5 | SDLKVEQVLTANEWQSTMVTVITDSLPDDPVGPLRRVNVESNVKYLPNGDYIRVANIKLFAQGSTAESTINISEKGRWEVSDNYLLVSPSEFKDISSSQSKDFSEAQLRLITQIFKLDAEQSRRIDVVNEKTLLLTSLNHGSSVLFKN | 65.07 |
| *V. fischeri* | ToxRp | Q56689 | PKSDAFVEVANYDNTPVFVPVNHPSIERWKPLIEQCTNFYNSKHTDSLKPIEVIATSGQPNQITLNYIHSEEHSDQSISVRLLIDQKGWIRYVDKQRAVTSIQTFITLNPANYLSNR | 45.74 |
|  | ToxSp | Q56690 | SDSKVEQLLISKEWQSMSTVRIDTDYEDMGPLKRADIKSNVVYLPNKTYSKSSTLTIYSGLADEIHPLTINVMETGNWDYSGDYLLIDPTEFKDVTASDNKNFSGSQKKLIMRVFRMDAQQSKRVDVVNDKTLLLTSLNYGSGILFSH | 48.61 |
| *V. tapetis* | ToxRp | A0A2N8ZBK2 | SPSPATFTTVGTFSDIPVKTPENHPDISKWIPSITQCVEVYIARHPNELAPVEVIATGGQNGQIALNYIHAPEHSSENVTLKLLAVQPDFNQVCTQ | 48.42 |
|  | ToxSp | A0A2N8ZBM0 | WLYWGSDAQVERVLTSREWQTRMVMRIYADTSTDVLEQDGEIGPLKKATIESNVKYLPNGTYLRVSRINLFSESVEASSIINVSESGNWEMSDNYLLIEPIEFKEISSNQRQDFTEKQLGVITQIFKMDSQQSRRVEIINTKALLMTSLDHGSAVFYSH | 58.50 |
| *V. nigripulchritudo* | ToxRp | U4KGF8 | PFLTYLGSQQKPAKFTTIAEVAGIPVRMPENHPSMQQSLPNIEKCIEHYASQHEGDLAPVQVIATGGQNGQIILNYIHSIHYSSENVTLRLFSTQPDFAQVCN | 44.79 |
|  | ToxSp | U4K7M4 | TQTSAKVENVLTSREWQSRMVTRLFIESDSEQDEVIGPLRKATIDSNVKYLPNGTYLRVSRINLYTGTHEKNTSSTINVSENGAWELSDNYLLIEPKEFKDISSNQGADFTDKQLKVITQIFKMDAQQSRRVEIINAKALLMTSLDHGSTVLYSH | 61.11 |
| *V. rotiferianus* | ToxRp | A0A6V7GT05 | TKPAESQFRQIAEFSGVPVMTPANHPQLMQWMPSIEQCVARYVENHTNDVMPVKVIATGGQGNKLVLNYIHDTDHSYENVTLRIFAGQNDPTDICK | 50.00 |
|  | ToxSp | A0A7Y3Z916 | SDLKVEQVLTSNEWQSTMVTVITDNLPDDTVGPLRRVNVESNVKYLPNGDYIRVANIKLFAQGSSAESTINISERGRWEVSDNYLLVTPSEFKDISSSQSKDFTDEQLRLITQIFRLDAEQSRRIDVVNEKTLLLTSLNHGSTVLFRN | 69.86 |
| *V. atlanticus* | ToxRp | B7VRN5 | PSQPTHRLNMSKVNDKTNTFTIESNDPQSKSLLDRLVSLPELNNSNFYITSNRTRIYVSCIYKENSTSASQSVNFSVDIKRPIAKVVNDIVHECQ | 20.00 |
|  | ToxSp | B7VII8 | GSDAKVERLLTQHEWQSKMVTLISDIKQEDSIGPLRKVELSSNAKYLPNGTYLRMSVVRLYSTQTAPANVINISETGQWDINDNYLLISPTEFKDVTSAERQDFSEEQLELITQVIKMDAEQSRRIDIINQKALLLTSLNHGSTVLFSN | 59.86 |

**Supplementary File 1e.**

**List of amino acid sequences of sensory domains of ToxRS proteins from different Vibrio species.** Table S5 includes UniProt ID of the used sequences as well as sequence identity values to ToxRSp from *V. cholerae*.

**Dali structural homology search of ToxSp bound to ToxRp**

**Hit Number / chain / Z-score / RMSD / aligned residues / total number of residues / identity % / description**

| 1 | 5KEW-B | 12 | 2.6 | 112 | 132 | 10 | VTRA PROTEIN | |  |
| --- | --- | --- | --- | --- | --- | --- | --- | --- | --- |
| 2 | 4zgf-A | 9.6 | 3.6 | 112 | 141 | 13 | UNCHARACTERIZED PROTEIN | | |
| 3 | 2kts-A | 8.4 | 2.9 | 97 | 117 | 7 | HEAT SHOCK PROTEIN HSLJ | | |
| 4 | 4l3r-B | 8.4 | 3.2 | 109 | 144 | 5 | UNCHARACTERIZED PROTEIN | | |
| 5 | 4u3q-B | 7.9 | 1.9 | 84 | 99 | 17 | 17 KDA LIPOPROTEIN | |  |
| 6 | 2mhd-A | 7.6 | 2.7 | 91 | 110 | 13 | UNCHARACTERIZED PROTEIN | | |
| 7 | 4iab-A | 7.5 | 4.3 | 97 | 142 | 9 | HYPOTHETICAL PROTEIN | |  |
| 8 | 6ohh-B | 7.5 | 2.7 | 98 | 128 | 12 | EF1P2_MFAP2B | |  |
| 9 | 5byp-A | 7.4 | 3.1 | 91 | 121 | 13 | PUTATIVE UNCHARACTERIZED PROTEIN | | |
| 10 | 3lhn-A | 7.3 | 2.6 | 91 | 108 | 9 | LIPOPROTEIN | |  |
| 11 | 3hty-A | 7.2 | 3.1 | 82 | 94 | 9 | HYPOTHETICAL PROTEIN BT_0869 | | |
| 12 | 4n7c-A | 7.2 | 2.4 | 95 | 174 | 7 | BLA G 4 ALLERGEN VARIANT 1 | | |
| 13 | 2la7-A | 7.2 | 2.5 | 99 | 145 | 8 | UNCHARACTERIZED PROTEIN | | |
| 14 | 2erv-A | 7.1 | 3.3 | 99 | 150 | 6 | HYPOTHETICAL PROTEIN PAER03002360 | | |
| 15 | 5jk2-A | 7.1 | 2.4 | 85 | 128 | 9 | TP0751 |  |  |
| 16 | 4rlc-A | 7.1 | 3.9 | 108 | 135 | 6 | OUTER MEMBRANE PORIN F | | |
| 17 | 2m4l-A | 7 | 3.1 | 84 | 99 | 8 | PROTEIN BT_0846 | |  |
| 18 | 4pr7-A | 6.9 | 3.4 | 89 | 171 | 11 | OLIGOGALACTURONATE-SPECIFIC PORIN KDGM | | |
| 19 | 2jw1-A | 6.8 | 3 | 91 | 115 | 12 | LIPOPROTEIN MXIM | |  |
| 20 | 5ha0-A | 6.4 | 2.4 | 89 | 156 | 7 | LIPOCALIN AI-4 | |  |

**Supplementary File 1f.**

**Structural homology search of ToxSp bound to ToxRp using Dali server (*Holm, 2022*).**
