# Supplementary material for: Vibrio cholerae’s ToxRS bile sensing system
Source: eLife. 2023 Sep 28;12:e88721. doi: 10.7554/eLife.88721 (PMC10624426; doi:10.7554/eLife.88721)
Supplement: Supplementary file 2. — List of all atom interactions of ToxRSp (pdb: 8ALO). The list was created using PDBsum online server (Laskowski et al., 2018). [file elife-88721-supp2.docx]

**Supplementary File 2 – ToxRSp atom-atom interactions**

*Vibrio cholerae's* ToxRS Bile Sensing System

N. Gubensäk^1*^, T. Sagmeister^1^, C. Buhlheller^1^, B. D. Geronimo^2^, G. E. Wagner^3,4^, L. Petrowitsch^1^, M. Gräwert^5^, M. Rotzinger^3^, T. M. I. Berger^1^, J. Schäfer^6^, I. Usón^7,8^, J. Reidl^1,9,10^, P. A. Sánchez-Murcia^2^, K. Zangger^3,9,10^ and T. Pavkov-Keller^1,9,10^*

1 Institute of Molecular Biosciences; University of Graz, Graz, Austria.

2 Laboratory of Computer-Aided Molecular Design, Division of Medicinal Chemistry, Otto-Loewi Research Center; Medical University of Graz, Graz, Austria.

3 Institute of Chemistry / Organic and Bioorganic Chemistry; University of Graz, Graz, Austria.

4 Diagnostic and Research Institute of Hygiene, Microbiology and Environmental Medicine, Medical University of Graz, Graz, Austria.

5 EMBL Hamburg; Hamburg, Germany.

6 RedShiftBio; Boxborough, Massachusetts, United States.

7 Institute of Molecular Biology of Barcelona (IBMB–CSIC); Barcelona, Spain.

8 ICREA, Institució Catalana de Recerca i Estudis Avançats; Barcelona, Spain.

9 BioHealth Field of Excellence, University of Graz; Graz, Austria.

10 BioTechMed-Graz; Graz, Austria..

*Nina Gubensäk, Tea Pavkov-Keller

Email: nina.gubensaek@uni-graz.at, tea-pavkov@uni-graz.at

**List of all atom interactions of ToxRSp** (pdb: 8ALO). The list was created using PDBsum online server (***Laskowski et al., 2018***).

Hydrogen bonds

--------------

<----- A T O M 1 -----> <----- A T O M 2 ----->

Atom Atom Res Res Atom Atom Res Res

no. name name no. Chain no. name name no. Chain Distance

1. 386 OG1 THR 256 A <--> 1791 O HIS 164 B 2.67

2. 386 OG1 THR 256 A <--> 1799 OG SER 166 B 2.72

3. 386 OG1 THR 256 A <--> 1799 OG SER 166 B 2.72

4. 460 ND2 ASN 266 A <--> 1799 OG SER 166 B 2.84

5. 505 O PRO 271 A <--> 1718 NH1 ARG 155 B 2.71

6. 546 ND2 ASN 277 A <--> 1724 OG1 THR 156 B 3.12

7. 557 N THR 279 A <--> 809 O LYS 41 B 3.07

8. 563 O THR 279 A <--> 818 N VAL 43 B 3.00

9. 560 OG1 THR 279 A <--> 1809 N VAL 168 B 3.01

10. 572 N ARG 281 A <--> 824 O VAL 43 B 2.87

11. 582 O ARG 281 A <--> 831 N LEU 45 B 2.91

12. 591 N VAL 283 A <--> 838 O LEU 45 B 2.90

13. 597 O VAL 283 A <--> 856 N THR 48 B 3.32

Non-bonded contacts

-------------------

<----- A T O M 1 -----> <----- A T O M 2 ----->

Atom Atom Res Res Atom Atom Res Res

no. name name no. Chain no. name name no. Chain Distance

1. 128 CD2 HIS 223 A <--> 1786 ND1 HIS 164 B 3.67

2. 127 NE2 HIS 223 A <--> 1784 CB HIS 164 B 3.76

3. 127 NE2 HIS 223 A <--> 1785 CG HIS 164 B 3.81

4. 127 NE2 HIS 223 A <--> 1786 ND1 HIS 164 B 3.33

5. 388 C THR 256 A <--> 1791 O HIS 164 B 3.76

6. 385 CB THR 256 A <--> 1791 O HIS 164 B 3.10

7. 385 CB THR 256 A <--> 1799 OG SER 166 B 3.66

8. 386 OG1 THR 256 A <--> 1790 C HIS 164 B 3.86

9. 386 OG1 THR 256 A <--> 1791 O HIS 164 B 2.67

10. 386 OG1 THR 256 A <--> 1796 N SER 166 B 3.57

11. 386 OG1 THR 256 A <--> 1798 CB SER 166 B 3.37

12. 386 OG1 THR 256 A <--> 1799 OG SER 166 B 2.72

13. 387 CG2 THR 256 A <--> 1799 OG SER 166 B 3.41

14. 390 N GLY 257 A <--> 1791 O HIS 164 B 3.19

15. 390 N GLY 257 A <--> 1784 CB HIS 164 B 3.89

16. 391 CA GLY 257 A <--> 1791 O HIS 164 B 3.59

17. 391 CA GLY 257 A <--> 1784 CB HIS 164 B 3.89

18. 393 O GLY 257 A <--> 1784 CB HIS 164 B 3.89

19. 444 CG2 THR 264 A <--> 1801 O SER 166 B 3.32

20. 444 CG2 THR 264 A <--> 1799 OG SER 166 B 3.39

21. 458 CG ASN 266 A <--> 1740 CD1 LEU 158 B 3.64

22. 458 CG ASN 266 A <--> 1799 OG SER 166 B 3.80

23. 459 OD1 ASN 266 A <--> 1740 CD1 LEU 158 B 3.87

24. 459 OD1 ASN 266 A <--> 1803 CA THR 167 B 3.81

25. 459 OD1 ASN 266 A <--> 1807 C THR 167 B 3.71

26. 459 OD1 ASN 266 A <--> 1809 N VAL 168 B 3.38

27. 459 OD1 ASN 266 A <--> 1811 CB VAL 168 B 3.89

28. 460 ND2 ASN 266 A <--> 1740 CD1 LEU 158 B 3.60

29. 460 ND2 ASN 266 A <--> 1799 OG SER 166 B 2.84

30. 479 CG2 ILE 268 A <--> 1689 CG1 VAL 152 B 3.76

31. 480 CD1 ILE 268 A <--> 1813 CG2 VAL 168 B 3.75

32. 504 C PRO 271 A <--> 1718 NH1 ARG 155 B 3.69

33. 505 O PRO 271 A <--> 1714 CD ARG 155 B 3.06

34. 505 O PRO 271 A <--> 1715 NE ARG 155 B 3.75

35. 505 O PRO 271 A <--> 1716 CZ ARG 155 B 3.64

36. 505 O PRO 271 A <--> 1718 NH1 ARG 155 B 2.71

37. 501 CB PRO 271 A <--> 1705 CD GLU 154 B 3.51

38. 501 CB PRO 271 A <--> 1707 OE2 GLU 154 B 2.77

39. 502 CG PRO 271 A <--> 1696 CG ASN 153 B 3.72

40. 502 CG PRO 271 A <--> 1698 ND2 ASN 153 B 3.79

41. 502 CG PRO 271 A <--> 1705 CD GLU 154 B 3.70

42. 502 CG PRO 271 A <--> 1707 OE2 GLU 154 B 3.04

43. 513 C GLU 272 A <--> 1718 NH1 ARG 155 B 3.43

44. 514 O GLU 272 A <--> 1718 NH1 ARG 155 B 3.40

45. 515 N VAL 273 A <--> 1718 NH1 ARG 155 B 3.53

46. 516 CA VAL 273 A <--> 1718 NH1 ARG 155 B 3.86

47. 522 N SER 274 A <--> 1718 NH1 ARG 155 B 3.21

48. 527 O SER 274 A <--> 1826 CB PHE 170 B 3.27

49. 527 O SER 274 A <--> 1827 CG PHE 170 B 3.42

50. 527 O SER 274 A <--> 1831 CD2 PHE 170 B 3.84

51. 524 CB SER 274 A <--> 1724 OG1 THR 156 B 3.54

52. 525 OG SER 274 A <--> 1724 OG1 THR 156 B 3.74

53. 528 N GLY 275 A <--> 1717 NH2 ARG 155 B 3.54

54. 529 CA GLY 275 A <--> 1828 CD1 PHE 170 B 3.66

55. 529 CA GLY 275 A <--> 1829 CE1 PHE 170 B 3.77

56. 531 O GLY 275 A <--> 1829 CE1 PHE 170 B 3.85

57. 531 O GLY 275 A <--> 1832 CE2 PHE 170 B 3.72

58. 531 O GLY 275 A <--> 1830 CZ PHE 170 B 3.52

59. 548 O ASN 277 A <--> 1831 CD2 PHE 170 B 3.61

60. 548 O ASN 277 A <--> 1832 CE2 PHE 170 B 3.54

61. 546 ND2 ASN 277 A <--> 1723 CB THR 156 B 3.79

62. 546 ND2 ASN 277 A <--> 1724 OG1 THR 156 B 3.12

63. 546 ND2 ASN 277 A <--> 1725 CG2 THR 156 B 3.18

64. 549 N ILE 278 A <--> 1812 CG1 VAL 168 B 3.85

65. 550 CA ILE 278 A <--> 809 O LYS 41 B 3.78

66. 555 C ILE 278 A <--> 809 O LYS 41 B 3.89

67. 555 C ILE 278 A <--> 1812 CG1 VAL 168 B 3.68

68. 556 O ILE 278 A <--> 1812 CG1 VAL 168 B 3.62

69. 552 CG1 ILE 278 A <--> 809 O LYS 41 B 3.42

70. 557 N THR 279 A <--> 809 O LYS 41 B 3.07

71. 563 O THR 279 A <--> 811 CA MET 42 B 3.24

72. 563 O THR 279 A <--> 816 C MET 42 B 3.60

73. 563 O THR 279 A <--> 813 CG MET 42 B 3.63

74. 563 O THR 279 A <--> 818 N VAL 43 B 3.00

75. 563 O THR 279 A <--> 824 O VAL 43 B 3.70

76. 560 OG1 THR 279 A <--> 1803 CA THR 167 B 3.47

77. 560 OG1 THR 279 A <--> 1807 C THR 167 B 3.68

78. 560 OG1 THR 279 A <--> 1809 N VAL 168 B 3.01

79. 560 OG1 THR 279 A <--> 1812 CG1 VAL 168 B 3.57

80. 561 CG2 THR 279 A <--> 1801 O SER 166 B 3.54

81. 565 CA LEU 280 A <--> 824 O VAL 43 B 3.54

82. 570 C LEU 280 A <--> 824 O VAL 43 B 3.67

83. 568 CD1 LEU 280 A <--> 824 O VAL 43 B 3.75

84. 568 CD1 LEU 280 A <--> 835 CD1 LEU 45 B 3.63

85. 572 N ARG 281 A <--> 824 O VAL 43 B 2.87

86. 573 CA ARG 281 A <--> 824 O VAL 43 B 3.81

87. 582 O ARG 281 A <--> 824 O VAL 43 B 3.52

88. 582 O ARG 281 A <--> 826 CA SER 44 B 3.33

89. 582 O ARG 281 A <--> 829 C SER 44 B 3.60

90. 582 O ARG 281 A <--> 831 N LEU 45 B 2.91

91. 582 O ARG 281 A <--> 838 O LEU 45 B 3.89

92. 574 CB ARG 281 A <--> 828 OG SER 44 B 3.61

93. 575 CG ARG 281 A <--> 828 OG SER 44 B 3.70

94. 576 CD ARG 281 A <--> 814 SD MET 42 B 3.86

95. 576 CD ARG 281 A <--> 828 OG SER 44 B 3.48

96. 577 NE ARG 281 A <--> 828 OG SER 44 B 3.81

97. 580 NH1 ARG 281 A <--> 814 SD MET 42 B 3.68

98. 580 NH1 ARG 281 A <--> 1609 OD2 ASP 142 B 3.77

99. 584 CA ILE 282 A <--> 838 O LEU 45 B 3.72

100. 589 C ILE 282 A <--> 838 O LEU 45 B 3.79

101. 591 N VAL 283 A <--> 838 O LEU 45 B 2.90

102. 592 CA VAL 283 A <--> 838 O LEU 45 B 3.73

103. 597 O VAL 283 A <--> 840 CA ILE 46 B 3.56

104. 597 O VAL 283 A <--> 845 C ILE 46 B 3.88

105. 597 O VAL 283 A <--> 847 N LYS 47 B 3.43

106. 597 O VAL 283 A <--> 856 N THR 48 B 3.32

107. 597 O VAL 283 A <--> 858 CB THR 48 B 3.46

108. 597 O VAL 283 A <--> 860 CG2 THR 48 B 3.19

109. 593 CB VAL 283 A <--> 838 O LEU 45 B 3.84

110. 594 CG1 VAL 283 A <--> 860 CG2 THR 48 B 3.76

111. 659 CG1 VAL 292 A <--> 833 CB LEU 45 B 3.64

112. 660 CG2 VAL 292 A <--> 849 CB LYS 47 B 3.09

113. 660 CG2 VAL 292 A <--> 850 CG LYS 47 B 3.87

Salt bridges

------------

<----- A T O M 1 -----> <----- A T O M 2 ----->

Atom Atom Res Res Atom Atom Res Res

no. name name no. Chain no. name name no. Chain Distance

1. 579 NH2 ARG 281 A <--> 1609 OD2 ASP 142 B 3.77

Number of salt bridges: 1

Number of hydrogen bonds: 13

Number of non-bonded contacts: 113
